# “I’m Wise to the Game”: How Inner-City Women Experience and Navigate Police Raids

**DOI:** 10.1177/15570851211005541

**Published:** 2021-04-02

**Authors:** Carolyn Greene, Marta-Marika Urbanik, Manzah-Kyentoh Yankey

**Affiliations:** 1 Athabasca University, AB, Canada; 2 University of Alberta, Edmonton, Canada

**Keywords:** gender, policing, qualitative research

## Abstract

Despite the plethora of research on inner-city policing, little is known about how women experience and make sense of involuntary police encounters. Based upon interviews with women who had their homes raided by police in Toronto’s inner-city, this paper explores how these marginalized women perceive, navigate, and resist normative gender expectations in their interactions with police officers during raids. Our findings demonstrate that women believed officers treated them according to gendered stereotypes, and in response, women strategically deployed gendered presentations in an effort to resist, negotiate, and temper anticipated raid related harms. However, participants’ positionality constrained their efforts.

## Introduction

In North America, police presence is heightened in many inner-city communities, subjecting residents to racial profiling, police abuses, and criminalization (e.g., Anderson, 1999; Brunson, 2007; Brunson & Miller, 2006a, 2006b; Cobbina 2019; Comack & Silver, 2008; Contreras, 2013; Gaston & Brunson, 2020; Maynard, 2017; Rios, 2011, 2017; Skogan, 2006; Weitzer & Brunson, 2009, 2013; Wortley & Owusu-Bempah, 2011). In response, some community residents draw upon strategies (e.g., police/area avoidance, verbal challenges, knowing one’s rights, and officer-specific tactics) to limit and mitigate police contact (Anderson, 1999; Goffman, 2014; Jones, 2014; Stuart, 2016; Urbanik & Greene, 2020; Weitzer & Brunson, 2009; Weitzer & Tuch, 2006). Despite this broad knowledge base, few empirical studies have examined more invasive and oft veiled police encounters, such as police raids. In Western countries, police raids often involve militarized police units forcefully and unexpectedly entering properties in search of a suspect and/or evidence (Cyr et al., 2020; Fisher, 2010; Kraska & Kappeler, 1997; Roziere & Walby, 2019). The March 2020 police killing of Breonna Taylor during a botched raid of her home in Kentucky, U.S.A. has ignited broader public criticism—largely led by the Black Lives Matter (BLM) movement—of residential search warrant execution. However, despite BLM’s continued advocacy and global protests since 2014 (see Lebron, 2017; Rickford, 2015), racially biased policing continues (Gaston et al., 2020; Roach et al., 2021; Ross et al., 2020).

Much of what we know about experiences of inner-city policing is rooted predominantly in men’s experiences (e.g., Brunson, 2007; Brunson & Miller, 2006b; Goffman, 2014). The relative paucity of research examining inner-city women’s experiences with police is curious because women—particularly racialized women—living in marginalized neighborhoods are also subject to heightened police surveillance, discrimination, and criminalization (Brunson & Miller, 2006a; Crenshaw et al., 2014, 2015; Maynard, 2017; Ritchie, 2017; Stuart, 2016). While scholars have examined women’s experiences with crime, victimization, and the criminal justice system (e.g., Adler, 1975; Burgess-Proctor, 2006; Cullen et al., 2014; Daly, 1992; Gartner & Kruttschnitt, 2004; Grundetjern, 2015; Hoskins & Cobbina, 2020; Jones et al., 2019; Kruttschnitt, 2013; Maher, 1997; Messerschmidt, 1995; Miller, 2001; Stearns, 2019; Steffensmeier & Allan, 1996), few studies have analyzed women’s involuntary police encounters outside of intimate partner violence situations (see Johnson, 2007; Saxton et al., 2021). Of those that have, most have documented women’s general perceptions of police, police trauma, harassment, microaggressions, and avoidance strategies during street encounters (Aniefuna et al., 2020; Brunson & Miller, 2006a; Brunson & Stewart, 2006; Bundy, 2019; Fine et al., 2003; Fox-Williams, 2019; Hitchens et al., 2018; Maynard, 2017; Rengifo & Pater, 2017; Wilson et al., 2020). One notable study—Goffman’s (2014, pp. 63–69) “On the Run”—documented how police treat women during police raids, illuminating officers’ techniques for coercing women’s cooperation. However, Goffman did not examine women’s perceptions of and/or responses to officers’ tactics. Thus, while some studies have explored how women use gender to *avoid* police attention/arrest (e.g., Jacobs & Miller, 1998; Maher, 1997), none examine how gender is used/performed *during* police interactions—a surprising omission since police encounters are a “gendered experience” (Brunson & Miller, 2006a, p. 538).

In unpacking gendered experiences, we acknowledge that people “do gender” within the context of everyday interactions, which shapes behaviour through “a complex of socially guided perceptual, interactional, and micropolitical activities” (West & Zimmerman, 1987, p. 126). Gender performances vary according to situated normative ideals and expectations about “masculinity” and “femininity” (West & Zimmerman, 1987), and are continuously renegotiated and redefined depending on context (Mavin & Grandy, 2011; Poggio, 2006). Accordingly, gender is a *calculated* performance; gendered presentations may be turned on or off depending on need and circumstance (Linstead & Pullen, 2006; Mavin & Grandy, 2011). Research has also attuned us to the intersections between gender, race, and class in the criminal justice system, such that women’s actions may be understood through a “. . . choice within constraint framework that emphasizes that everyone, regardless of gender, actively navigates social environments and access to resources to creatively construct pathways” where “. . . choices are limited by intersecting inequalities of opportunities and constraints” (DeCoster & Heimer, 2017, p. 17). While scholars have examined the intersectional aspects of women’s crime and victimization, the relative paucity of research on the gendered elements of police encounters means we continue to know little about how gender shapes police-initiated interactions within marginalized communities (Cobbina et al., 2019, p. 282).

To address this knowledge gap, we position this paper at the nexus of an intersectional, multiracial feminist approach that explores women’s agency during involuntary police encounters (see Burgess-Proctor, 2006; Daly, 1992, 2010). Through this lens, we unmask how women living in Toronto’s inner-city perceive, navigate, and respond to police interactions during raids. We illustrate participants perceived police held stereotypical, heteronormative views about their womanhood and/or motherhood identities and allegedly used these assessments to determine how they would treat the women. We then demonstrate that as a consequence, women saw “doing gender” (West & Zimmerman, 1987)—specifically by performing the “good woman”/“good mother”—during these interactions as an important resource that could sometimes buffer police treatment and mitigate raid-related harms. Drawing from Miller (2002) and DeCoster and Heimer’s (2017) instructive work, we provide insight into the situational contexts shaping women’s involuntary police encounters. We demonstrate our participants-within the constraints of multiple intersecting inequalities and identities-strategically crafted, shifted, and/or abandoned gendered performances in response to perceived police expectations and treatment. Accordingly, we expand our understanding of women’s agency in an under-explored context.

## Methodology

### Setting

Our study is based in Regent Park and St. James Town, two inner-city Toronto (Canada) neighborhoods located a few blocks away from each other. Established in the 1950s, both neighborhoods are economically mixed, though they are most commonly associated with social housing, with some residents likening them to American “ghettos.” Regent Park is Canada’s oldest social housing development, home to approximately 11,000 residents and currently undergoing ‘revitalization.’ Built a few years later, St. Jamestown is the country’s most densely populated urban area, with some 18,615 residents. Both communities are generally low income and racially/ethnically diverse. In 2016, 60% of Regent Parkers under 17 years and 40% of adults (18–64 years) lived in poverty, with 70% of residents identifying as visible minorities (Statistics Canada, 2018). Similarly, in St. James Town, 40% of residents lived at or below the federal poverty line (see Statistics Canada, 2021) and 67% of residents were visible minorities (City of Toronto, 2018). Many Regent Park and St. James Town residents experience notable challenges and stigmatization, oft having to navigate compounding risks to their wellbeing, including gun violence, victimization, and police abuses (Urbanik, 2017; Urbanik & Greene, 2020). Simultaneously, local media accounts have been dominated by “discourses of vilification” (Wacquant, 2008, p. 240), portraying these neighborhoods as lawless, and rarely conveying how structural oppression shapes residents’ lived realities. Apart from further stigmatizing community denizens, “external representations” (Purdy, 2005, p. 524) have also consistently failed to acknowledge residents’ resilience in the face of social, economic, racial, and political marginalization, young residents’ efforts at upward mobility, strong community ties and an unwavering commitment to activism.

### Data Collection and Analysis

The findings presented herein are gleaned from a larger study exploring resident perceptions and experiences of police in three Toronto (Canada) inner-city neighborhoods: St. James Town, Regent Park, and Blake-Boultbee. Between 2016 and 2018, we conducted 45 interviews and spent long hours with participants in their respective communities. In 2020, we re-interviewed nine participants to further bolster our data. REB approval was granted by Athabasca University (22,200) and the University of Alberta (Pro00074759). A notable subset of participants—35—reported experiencing a police raid. While men and women reported similar perceptions of latent police misconduct and violence during police raids (see Urbanik & Greene, 2020), analysis for this paper revealed women’s experiences were markedly different from their male counterparts on the basis of perceptions about police deployment of stereotypically gendered assumptions, treatment, and threats. As such, we wanted to explore women’s unique experiences in greater depth, unmasking women’s gendered experiences and situated agency during under-studied police encounters.

This paper draws upon interviews with the 12 female participants (21–54 years) from Regent Park and St. James Town who had their home raided by police. Several participants reported being raided multiple times, sometimes years or a few days prior to our interview, and all reported officers on scene were predominantly male. Five women were Black, four were white, three were Indigenous, and all but two were mothers. Eleven participants lived in social housing, all were low-income (living below/near Canada’s Low-Income Cut-Off (LICO)), and 10 reported participating in crime (e.g., managing trap houses/drug trafficking/weapons storing). All had long histories of police contact generally commencing in early adolescence. Each reported their homes were raided as part of investigations relating to male affiliates (romantic partner/child) suspected of drug trafficking, weapons, robbery, and/or homicide offences. None reported being the suspect of the criminal investigations triggering the raid.

We relied upon our existing connections in these neighborhoods to facilitate participant recruitment. This included ethnographic research in Regent Park since 2013 for Urbanik (see Urbanik, 2017), and lifelong personal relationships for Greene. Strong rapport prior to conducting formal interviews is critical (Bucerius, 2014), and our long-standing relationships likely increased participants’ comfort in sharing their experiences with law enforcement, particularly in an environ of pervasive distrust of police. These relationships also allowed us to spend long hours with participants in their respective neighborhoods, challenge and/or triangulate narratives, and to deploy snowball sampling, where some participants recruited others into the study, which diversified our sample (see Urbanik & Greene, 2020 for more information). Interviews were audio-recorded, transcribed, and anonymized. In order to protect participants’ identities, all assigned names are pseudonyms and we have altered some details. We interviewed participants in locations of their choosing, with most interviews occurring within their homes. Interviews lasted 20 minutes-several hours (averaging 1 hour), and participants were compensated $30.00 for their time. We adopted a semi-structured interview guide and pursued new themes as they emerged until we reached thematic saturation (Guest et al., 2006; Small, 2009).

Inter-coder reliability tests were completed by having each author and two research assistants independently identify main codes and sub-codes for a set of five interviews. These checks resulted in approximately 90% agreement. After we completed initial coding, we followed best practices and reviewed all transcripts to ensure all relevant data were captured and explored in relationship to these codes (Braun & Clarke, 2006). We then reviewed the transcripts again to ensure that the organizational and substantive categories accurately reflected participants’ perspectives and thoroughly represented the data. We conducted a second round of reliability checks which produced agreement on the quotes’ representation of identified codes and sub-codes.

Throughout all stages of data collection, we continually analyzed interviews, submersing ourselves in the data by frequently re-reading transcripts and identifying initial free codes. With a team of research assistants, we primarily deployed inductive thematic analysis to guide theme and comprehensive coding scheme development (Charmaz, 2011), though we did employ elements of deductive analysis (e.g., focus on resistance and agency) (Braun & Clarke, 2012, pp. 58–59).

Through “open coding,” we identified six broad “organizational categories” (Maxwell, 2012, pp. 107–108), including: perceptions of police, direct experiences, vicarious learning, and neighborhood-specific knowledge. We also identified 17 “substantive categories,” which included: gendered discrimination, treatment of non-suspect during raid, racism, police violence/misconduct, and positive police interactions. We then carefully examined and compared relationships between codes, which assisted us in developing theoretical concepts.

## Findings

Gender and its intersections with other structural inequalities such as race and class permeated many aspects of women’s raid experiences. Whereas male accounts of police raids centered upon officers’ verbal and physical aggression (Urbanik & Greene, 2020), female participants perceived police deployed gendered stereotypes to ‘manipulate’ or coerce them into cooperating with law enforcement. One participant, Lysandra (Black-30 years) succinctly articulated these differences: “My bitterness lies with being manipulated as opposed to being beat up.” These stereotypes were inherently tied to two, often intersecting characteristics: women’s connections to male affiliates (usually partners) and motherhood identities. Participants responded to these perceived police tactics by skillfully engineering dynamic strategies revolving around gendered presentations in efforts to buffer their raid experiences and mitigate anticipated raid-related consequences.

### Women as ‘Victims’ of Criminally Involved Men?

Participants widely believed police used their relationships with male suspects as leverage to elicit information about criminal activities and/or coerce women’s cooperation. For example, Monique (white-28 years) recounted what she perceived as police intimidation attempts when they raided her home as part of a robbery and homicide investigation targeted at her partner. She recalled officers attempting to set her against her boyfriend by asserting she was naive to the seriousness of the situation: “I could tell they’re trying to scare me. . .they’re trying to act like they’re on my side. ‘Please don’t make this mistake, you’re gonna ruin your life for this guy’.” Another participant, Gabby (white-41 years), an independent drug trafficker, recounted police yelling while they raided her home looking for drugs and firearms they suspected belonged to her boyfriend:

“‘Do you know your boyfriend is a fucking drug dealer? He’s a piece of shit, he’s a scum bag’. . .Just repeating ‘you know you’re with a drug dealer, you know you’re with a bad guy, he’s a piece of shit, he sells drugs, why would you be with a person like that?!’. . . Trying to get me to admit to things.”

Similar to Monique, Gabby perceived police tried to create distance between her and her partner by implying she must be unaware he is a “piece of shit” and a “bad guy,” so she would “admit to” [inform on] his criminal activities. Both Gabby and Monique believed officers attempted to manipulate them by presenting them as naive white women duped by their Black male partners, so they would cooperate in the investigation.

Gabby further described what she perceived as officer attempts to humiliate her into cooperating. In recounting what happened after unlocking her safe as per officer demands, she explained: “They were laughing at the fact I had a vibrator. ‘Oh, you gotta big Black one?’ and making comments about the size of the vibrator.” Apart from being degrading, Gabby insisted these remarks were racially motivated: “The whole stereotype of Black men-and the comments about the dildo had racial undertones because he [boyfriend] is Black and I am white.” Such associations are rooted in racist stereotypes of Black male criminality and sexuality, which officers sometimes direct at white women in interracial relationships (see Hitchens et al., 2018, pp. 35–36). When we asked Gabby why she believed officers treated her this way, she responded: “I guess they were hoping to shock me into saying ‘Oh my God, now that you say that I DO remember’.” Gabby’s allegation is consistent with previous research documenting police routinely pressuring loved ones in marginalized neighbourhoods, including family members, friends, and girlfriends to inform on men’s criminal behaviours (Goffman, 2014; Natapoff, 2009).

Participants also recounted how police used them as leverage against their partners. To illustrate, Jodie (Indigenous-35 years), whose home was raided due to her boyfriend’s alleged drug trafficking, accused police of planting drugs and charging her to coerce her into informing on her partner. She explained: “I think they[police] put it[planted crack] there! ‘Cause otherwise they couldn’t charge me, and they needed[me to talk]. I think they were gonna try to use me as weight against him.” Monique, likewise described officers trying to use her as “a tool” to gain her boyfriend’s cooperation:

“They came in and they said they followed him[boyfriend] for a month, they looked at our cell phone records and they [police] realized nothing mattered to him but me. They needed me to pressure him, they wanted him to confess. They get me, take my mug shot, they go up to the jail he’s in, they slam it on the table, and say ‘We have Monique! We have Monique!’. It was a game to them. They know I had nothing to do with it, it wasn’t in the interest of justice.”

Many of the women in our study reported being subjected to superfluous harms, such as officers berating their relationship choices, planting evidence, and nefariously charging them with crimes, all in an effort to gather evidence and bolster cases against male suspects.

### Playing and Resisting the ‘Victim’ Identity

Our data also reveal participants perceived police held stereotypes of women as docile and naive, as Gabby elucidates: “They[police] have the same stereotypes in their heads as most of society. They accepted that I was a victim, unknowingly pulled into this situation by a bad Black drug dealer which was basically the role I fell into.” Whilst our participants emphasized these stereotypes were incongruent with the types of women they “actually” are, they recognized adhering to such stereotypes could sometimes buffer how police treated them. Kessler and McKenna (1978) highlight the important the role gender attributors (e.g., male police officers) play in shaping how gender is performed and argue this role is equal to gender displayers (e.g., our female participants). This theme is consistent with our findings. For example, consider Gabby’s thought process upon realizing police were raiding her home:

“Once I had been woken up and realized what was happening, I made the conscious decision to either go the sad victim route or the defiant route. And I decided to go the victim route because I knew there was nothing[no contraband] in the house and I had a child in the house. I pretended I was traumatized and cried. That’s not me. I’m wise to the game. They [police] thought they could scare me into doing or saying whatever they wanted and so I played into that. But I didn’t do or say anything I didn’t want to. I wanted to try and manipulate them at the same time.”

Far from passively accepting officers’ real or perceived tactics, the women in our study crafted dynamic strategies—within structural and situational constraints—in an attempt to resist police power and “manipulation” and mitigate raid-related consequences. Since Gabby perceived police *would* and *did* ascribe heteronormative gender ideals to her and believed they could “scare” her into “doing or saying whatever they wanted.” She adjusted her gendered performance accordingly. She swiftly feigned being a “sad victim” which she described as a strategic performance aimed at “manipulating” the police back designed to influence the interaction. Consequently, she played into perceived police expectations by crying and “pretending” she was traumatized, intentionally manufacturing/accentuating traditional or “emphasized” femininity—a dominant and idealized form of womanhood, defined around compliance, subordination to men, and accommodating men’s interests and desires, including power and control (Connell, 1987, 1995). Critically, Gabby was adamant that the “sad victim” role was an act she conjured up because she is “wise to the game.” Gabby explained why performing normative femininity could mitigate anticipated harms:

“I was angry. I did want to tell them to get the fuck out of my house but I was smart enough to know that I couldn’t. So, I decided to play the victim role which would cause the least amount of damage. Almost like a woman getting beaten, laying on the floor in the fetal position to stop getting hurt. An abused woman like me knows that if you keep fighting back you’re gonna get hurt worse. Whereas if you lay on the floor and you huddle, you may still get hurt but it’s gonna be less, and it’s gonna cause less bruising and damage to your face. . .I think just from being a woman I knew that dealing with men that were in positions of power that I would be able to maybe ease up my situation by playing the victim.”

Gabby’s declaration she was “smart enough to know” she could not freely express herself and overtly resist police efforts without risking additional harm demonstrates participants’ gender enactments during such encounters were shaped by “choice within constraints” (DeCoster & Heimer, 2017, p. 15). As troubling as Gabby’s analogy of her experience with police mimicking domestic abuse is, it reflects her understanding of the inherent power imbalance between her and the officers searching her home; while officers held ultimate power, participants situationally assessed and responded to anticipated police behaviours in an attempt to covertly influence police interactions and possible outcomes.

### Strategic and Dynamic Gender Presentations

Crucially, participants described an ongoing, dynamic process of situational evaluation and modified their gendered presentations accordingly. Monique, who has been raided multiple times and considers herself “experienced” with police “tactics,” depicted drawing upon this knowledge during a recent raid where officers found money in her apartment they suspected were proceeds of crime. Since some inner-city Toronto residents believe officers “steal”—unlawfully seize—money during police raids (see Urbanik & Greene, 2020), Monique recognised she needed to act quickly to preserve her cash, so she feigned vulnerability: “They even took out the $500 and I started bawling. I’m like, please don’t take that then I can’t afford my rent. . .And they actually ended up leaving my money there.” Monique insisted this pitiful position was a performance; she did not need the cash for rent. As our data reveal, women in our study adhered to stereotypes of women as docile, weak, and vulnerable when they perceived it would benefit them. Their efforts to enact emphasized femininity in the hopes of receiving better police treatment are consistent with the chivalry hypothesis, which finds women who adhere to traditional, heteronormative gender expectations are treated more leniently (Branscombe & Owen, 1991; Franklin & Fearn, 2008; Koons-Witt, 2002; Koons-Witt et al., 2014).

However, contrary to accepting being naive victims of police tactics and/or abuses, the women in our study emphasized their knowledge and agency as a source of power in addressing perceived police manipulation. Participants’ gendered performances were tools based upon “complex strategic combinations of compliance, resistance, and cooperation” (Connell, 1987, p. 184). For example, while police continued to pressure Monique, she claimed she saw through their efforts: “You’re trying to get me to incriminate myself-you’re trying to put myself at a scene of homicide-I’m not stupid! They finished their whole little speech, ‘We know you’re a good person’- their whole bullshit. It was insulting to my intelligence.” Monique even found officers’ attempts amusing: “I’m dating a criminal! You’re trying to criminal a criminal! (laughs).” Similarly, Chrissy (Black-21 years-no children) was keen to affirm, despite officers trying to enter her home without a warrant, she knew the limits of police powers: “I’m not stupid! I know my rights; I’m not gonna be fucked around with.”

While some of our participants enacted “emphasized femininity” when they believed it to be advantageous, they changed their performance when this gender script was no longer beneficial. To illustrate, Monique described her initial acceptance of the search, and articulated switching her response and reverting to the type of woman she “really is” to address perceived police manipulation:

“Okay I get it; you guys have to search the house. . .They’re like ‘We’re gonna have a talk now’, ‘you’re a witness right now’, ‘it depends on how our conversation goes’[charges possible]. The insinuation was, ‘You’re gonna tell us what we want to know, and we’ll leave you alone’[avoid charges]. Which I know they weren’t gonna do, because I already had a warrant for my arrest. If I had been stupid enough to talk in that moment, I would’ve roasted myself anyway. I said to them, ‘No, I don’t really want to have a conversation right now’. . .Then they tried to make it the illusion that I was free to go and this was at my own will. But it wasn’t. I was smart enough to realize that. They didn’t know who they were dealing with.”

Likewise, Naomi (Black-21 years), illuminated how women’s response strategies shift depending on situational context. Police previously raided her home a number of months prior, arresting her partner. When describing a subsequent search while her boyfriend was incarcerated, she recounted:

“They’re [police] like ‘Where is this guy? We’re looking for him!’ and they started calling out his name. I was like ‘Um, I don’t know what you’re talking about’. I was just playing stupid. ‘I don’t know what you’re talking about’. They’re like ‘What car did he just leave in?’. And after that, I was like ‘Ok, I’m done playing this shit with you, I know who the fuck you guys are talking about. Like, this nigga’s in jail! Like this nigga’s been in jail since you guys came to my house how many fucking months ago and arrested him.”

Similar to Monique, while Naomi initially “played stupid” and did not overtly resist police efforts, she eventually re-evaluated the situation, dropped this presentation, and adopted a more adversarial position once she realized her passive stance was ineffective. While women performed “emphasized femininity” when they believed it would hinder harms, participants maintained they dropped these enactments when they were futile.

### Motherhood and Household: Vulnerability and Resource

Throughout our interviews, participants also reported what they perceived as police using motherhood status to simultaneously demean and attempt to coerce them into compliance. Several women—and specifically, Black mothers—described officers questioning whether they were “good” mothers and positing they were reproducing crime via their children. Taylor (Black-52 years) recounted police knocking on her door, demanding to search her home:

“They said, ‘One of your sons was seen with a gun’. I’m like ‘My sons don’t have guns’, and I think one of the cops actually admitted that it might be a water gun. So, I picked up the little water pistol, and said ‘Are you talking about this?’ And I said ‘Don’t you have something better to do?’ You know? And they were just really rude, really insulting. They wanted to search the house, I said ‘You can’t search the house, you don’t have a warrant’. And one of them said, ‘You know, women like you shouldn’t be allowed to breed, you know you just breed criminals. . .sick of you single mothers with kids running amok.’”

Similarly, Lysandra reported police telling her to “stop having children because every child you have will be a criminal.” The dominant ideology of motherhood carries expectations that mothers assume full responsibility for their children and are consequently blamed when children cause problems (Chan & Chunn, 2014; Dow, 2016). Black mothers in particular are often accused of reproducing “deviance” by “. . .transfer[ing] a deviant lifestyle to their children that dooms each succeeding generation to a life of poverty, delinquency, and despair” (Roberts, 1997, p. 8). Apart from its racist and classist underpinning, blaming mothers for children’s involvement in crime obscures marginalized mothers’ struggles in navigating racism, poverty, violence, and criminalization in disadvantaged neighborhoods (Crenshaw, 2013).

When mothers attempted to resist police interventions, some reported police threatening to report them to child welfare authorities to coerce their compliance and cooperation (see also Goffman, 2014, pp. 65–66). In these instances, mothers felt exceptionally vulnerable recognizing this could—and for many racialized women in Canada often does—result in welfare authorities taking away their children (Government of Canada, 2020; Maynard, 2017, pp. 186–207). These concerns were likely exacerbated by the fact that police and child welfare systems are sometimes perceived as analogous (Clarke, 2012, p. 239), and poor Black parents are more likely to be labelled as neglectful (Maynard, 2017, pp. 196–204). Naomi described police repeatedly entering and searching her home over a period of a few weeks, and explained what happened when she refused them entry one night:

“I’m not opening my door. . .You’re not coming to my fucking house, I don’t care, like you know? So, it was like after that, basically they threatened me with the Children’s Aid. . .I opened my door. They did their little search. They see nobody was there. That was the only time that I actually opened my door, cause I’m like ‘Bro, like I don’t need the Children’s Aid involved in my life!’.”

Here, Naomi makes clear that while she initially resisted police efforts, officers’ threats to contact Children’s Aid compelled her to relent. In these situations, the women in our study perceived police exploited participants’ vulnerability to pressure them into compliance. Naomi’s experience highlights women’s dynamic assessment and response to police actions is situational *and* constrained by positionality.

Once police entered their apartments, participants perceived police made inferences about them based upon two distinct, albeit often related, components—their motherhood capabilities and condition of their homes. According to women in our study, these assessments informed how officers treated them during raids. To illustrate, consider Lysandra’s description of the raid onset:

“They[police] bust through the door. . .I didn’t know who it was. I honestly ran straight towards them, and when I did that, they slammed me on the floor. . .I’m screaming, like ‘My son’s in the room! My son’s in the room!” And when they realized like there’s a baby in the room, he[police officer] kind of let go. But he put me in handcuffs for a second, and he told me to sit on the couch. . .He’s like ‘I’ll un-cuff you, but you can’t go nowhere’. And I said, ‘I’m not going to go nowhere, as long as you let me go, cause I don’t want my son to see that mom’s in cuffs’ you know?”

She provides additional context, comparing her most recent raid experience to a previous one:

“The way they treat me was different. Like they came in really aggressive, but when they seen that it’s a mom and a child, they switched their whole perspective. . .and I believe because I look more white than a Black person, you know? So, when they seen that, they’re like ‘Ok, so it’s a mom, a young mom.’ They were a little rude in the beginning, but after that they’re more like, ‘Wow, in all my years, I’ve never seen a mom run for their child, ever. Like, no matter how much raidings I’d been in, no mom has ever ran to their child, they’re mostly petrified’. So, they’re more like talking to me in a nice manner.”

According to Lysandra, officers “*switched their whole perspective*” and afforded her better treatment during the most recent raid because she successfully presented as a “good mother” (see also Hardesty & Black, 1999; Hoskins & Cobbina, 2020; Stearns, 2019). Conceptions of the “good mother” position women as caring nurturers readily available to care for their children, irrespective of circumstance (Collins, 2000; Kline, 1992; Krane & Davies, 2007; see also Dow, 2016). By rushing at police and trying to prevent her son from seeing her in handcuffs, Lysandra successfully fulfilled this expectation.

This account also attunes us to the nuances and intersectionality shaping women’s experiences with police in marginalized neighborhoods. Despite being a Black woman, Lysandra claimed her racial presentation as “more white” than Black made officers more likely to see her as a “caring mom,” consequently treating her better. This is consistent with arguments that white mothers are afforded special privileges because of their “ideal” femininity (Collins, 2000; Kline, 1992; Pyke & Johnson, 2003; Skeggs, 1997), whereas Black women are often portrayed and perceived as “welfare queens” and negligent mothers (see Collins, 2000; Maynard, 2017). For decades, Black feminist scholars have argued the feminine ideal is inconsistent with Black women’s experiences, in part, because their social positioning has necessitated independence, strength and resilience—traits that are incompatible with hegemonic femininity (Collins, 2000, 2006; Davis, 1983; Jeffries, 2015). Further, Lysandra’s perception is tied to colourism—the privileging of light-skinned racialized minorities over those who have darker skin (Hunter, 2007)—which has also affected other Black-Canadians’ police encounters (see Creese, 2020, p. 138).

However, participants contended officers’ perceptions of “good mothers” could not be divorced from officers’ perceptions their apartment’s condition. This is perhaps unsurprising as “good mothering” is oft intimately related to dominant white middle-class ideals of a ‘proper’ home (Kline, 1992). Mothers in our study believed showing concern for their children and keeping an orderly home softened how police treated them and their property. Below, Lysandra describes officers conflating her child rearing abilities with what they observed in her apartment:

“They[police] just basically threw everything everywhere, like you know?. . .Broke up some stuff. But not as bad [as the previous raid]. But as I said, they treated me a little better because they seen that I was actually like a caring mom. Like, I really cared about my kid. And they’d seen that my house was clean. Even when the guy[police officer] came in and opened the fridge, he’s like ‘Wow, there’s food in your fridge’, you know? So, they judged me for what they’d seen.”

Natalia (white-36 years) shared a similar narrative, suggesting her raid experience was shortened and officers caused less property damage than they otherwise may have because of her apartment’s presentation: “I think they[police] left early because they saw the type of house they were in. It was well kept, it looked like a suburban home. I care a lot about my child and his safety. His room was clean and well decorated.” Natalia believed her suburban-looking home signified her rectitude to officers, distancing her from families in other “types of houses” (see also Bell, 2016, p. 334). Both Natalia and Lysandra perceived home cleanliness and a displayed commitment to their children communicated an idealized notion of motherhood to officers, thereby protecting them and their property from additional harms (see May, 2008).

Previous research has found marginalized mothers enact motherhood as a resource in various settings, including prisons (Aiello & McQueeney, 2016; Barnes & Stringer, 2014), gangs (Miller, 2001; Moloney et al., 2011), illegal drug economies (Grundetjern, 2018; Miller et al., 2015), and sex work milieus (Maher, 1997). Similarly, women in our study sometimes enacted motherhood as a resource during police raids, emphasizing their presentation as “good mothers” in efforts to mitigate raid-related harms. For example, consider Natalia lying to police at the raid’s onset about her son having a disability:

“My first concern [when raid started] was my son. As soon as I put my pants on, I came out. I was like ‘Listen I have a son in this house, he has developmental issues’, which isn’t necessarily true. But, I didn’t want him to be upset. So I said what I have to say to get them [police] not breaking everything and smashing shit and screaming. . .At that time, it was the only thing I felt like I could do to protect my child from what I thought and knew they [police] were about to do [damage the home].”

Notably, she clarified her perspicacity was motivated by efforts to both preserve her son’s wellbeing *and* discourage officers from “breaking everything and smashing shit.” Recognising police often damage homes/property during raids, Natalia did “*the only thing*” she felt she could to prevent these superfluous harms, in the face of few other options.

Some women also used motherhood to prevent officers from seizing money during raids. For example, Shay (Black-32 years) was worried police would “steal” the $3000 they located in her apartment. In an effort to discourage officers from seizing it, she contrived a heartfelt explanation:

“They[police] searched our house, they ripped apart everything. We had money saved up, and they were gonna take the money. But I told them it was for my daughter’s birthday. But they were like, ‘That’s a lot of money, you know, for your daughter,’ But then they didn’t take nothing.”

During our interview, Shay clarified the cash was not for her four-year-old. While women’s performances of “good mother” reinforced prevailing normative assumptions about femininity, race, and class, they also underpinned how women challenged structural oppression. Paradoxically, our data reveal while police used women’s motherhood status as a *tool of police power*, participants situationally enacted their motherhood as a *tool for resisting* police power.

## Discussion

This exploratory study provides insight into how gender shapes police-resident encounters during police raids, with a focus on how women *perceive* and *manage* gendered interactions, expectations, raid-related harms, and harm-mitigation strategies. The women in our study perceived officers held stereotypical expectations of women as naïve and manipulatable. Our participants maintained officers “judged” the types of women/mothers they were, drawing upon intersecting, structural inequalities of their gender, race, class, and place (see Burgess-Proctor, 2006; Crenshaw, 1991), and used these judgments to guide how they treated them. In doing so, officers exploited women’s existing vulnerabilities by threatening/utilizing superfluous and *gendered* maltreatment.

However, our participants not passive victims to these police tactics. Their positionality and associated life struggles necessitated they approach all challenges—including police misconduct—with resourcefulness and tenacity. Accordingly, the women in our study relied on their existing knowledge and previous experiences with police to predict, respond to and resist, anticipated police abuses through adopting noteworthy counter-techniques. They quickly and carefully weighed their options amidst the circumstances to determine which situational armor would be most effective at hindering police misconduct and abuse of power. Sometimes, feigning emphasized femininity protected them, though women ‘un-did’ this gendered script when this performance was unbeneficial. Their techniques could be heavily strategic and goal oriented (*playing* the ‘victim’) or more routinized (*emphasizing* the “good” mother), though each were intended to buffer women’s raid experiences and persuade officers to treat them better.

Our work unmasks how marginalized women exert agency in an under-explored and often veiled context. Participants’ accounts reveal the “. . .restraint, dynamic and potentially transformative aspects of agency” in their structured responses during police raids (Miller, 2002, p. 440). The women in our study *looked for*, *created*, and *seized* opportunities to exercise their agency to shape/alter raid experiences. Critically, women’s narratives suggest that a multiracial feminist criminological paradigm attunes us to the importance of considering how women’s positionality—with attention to intersecting inequalities and identities—influences and constricts agency. Simultaneously however, though their positionality made them more vulnerable to police abuses than other women, their strategies were also shaped and informed by the “tool kit” or repertoires they had access to (Swidler, 1986). Given our participants’ life circumstances and direct and vicarious experiences with police, they had more expansive tool kits for navigating police interactions than other women (Lamont & Small, 2008). Though officers held ultimate power in these interactions, participants worked to influence how police exercised their discretion whenever possible. However, as “any theory of agency must be placed in the context of structural, institutional, or intersubjective constraints” (McNay, 2000, p. 20), participants’ gendered performances must be understood within the context of the structural constraints they faced while “bargaining with patriarchy” (Kandiyoti, 1988, p. 275).

As our data reveal, participants recognized they had few alternatives in terms of gender presentations in their dealings with police. Their positionality limited their options and they were aware that poorly selected and executed gendered presentations could have deleterious consequences, including accrual of criminal charges, child welfare interventions, and (un)lawful property/money seizure. In this sense, women understood officers would hold them “accountable for their gendered performances” (Miller & Carbone-Lopez, 2015, p. 695; see also Messerschmidt, 1995, p. 172). Accordingly, our participants’ actions must be viewed as “choice within [the] constraints” of structural inequalities (DeCoster & Heimer, 2017, p. 15) and perceived expectations of gender attributors—in this case, police officers (Kessler & McKenna, 1978). For the women in our study, these factors influenced how “femininity is defined, enacted, and achieved” (DeCoster & Heimer, 2017, p. 15) during police raids.

We interviewed 12 women as part of this exploratory study. Though this is an obvious limitation, the secrecy with which law enforcement officials execute raids and residential search warrants and associated stigma of being subject to such an invasive policing practice can impede locating and recruiting participants. These obstacles have likely limited empirical work in this area, with only a few studies examining citizens’ experiences with police raids (Goffman, 2014; Urbanik & Greene, 2020). This small sample size restricts our ability to draw conclusions about how participants’ positionality (race, ethnicity, age, etc.) affected their perceptions of and experiences with officers. Further, our findings may not be representative of women’s experiences in the respective neighborhoods or other field sites, as our data only speak to the experiences of this *particular* and rather *unique* subset of women, highly experienced in navigating police interactions. Despite these limitations, the richness of participants’ accounts has begun to unmask how women perceive and experience invasive police encounters that have routinely escaped empirical scrutiny.

Future research should build upon this foundational work and examine other structural and situational contexts that may affect perceptions of and experiences with police raids and other invasive policing practices. We also urge scholars examining police-community relationships to depart from the traditional default focus on men (see Maynard, 2017). More scholarly attention should be devoted to illuminating and analyzing women’s unique experiences with and strategies for dealing with law enforcement officials, both during and beyond traditional street encounters (in particular, during interactions within private residences). Future research on search warrant execution/raids should examine whether/how officer gender, pre-employment socialization, race/ethnicity, LGBTQ2+ identities, and/or neighborhood impacts these gendered encounters.
